# Therapeutic potential of a novel semi-synthetic-sulfated-polysaccharide to suppress inflammatory mediators in *P. gingivalis* LPS stimulated human monocytes/macrophages

**DOI:** 10.1186/s12950-021-00292-6

**Published:** 2021-09-04

**Authors:** Ying Gu, Veena Raja, Hsi-Ming Lee, Houlin Hong, Glenn Prestwich, Maria E. Ryan

**Affiliations:** 1 Departments of General Dentistry, School of Dental Medicine, Stony Brook University, Stony Brook, NY 11794 USA; 2 Department of Oral Biology and Pathology, School of Dental Medicine, Stony Brook University, Stony Brook, NY 11794 USA; 3 Program in Public Health, Stony Brook Medicine, Stony Brook University, Stony Brook, NY 11794 USA; 4 Department of Medicinal Chemistry, The University of Utah, Salt Lake City, UT 84112 USA; 5 Health Sciences Spokane, Washington State University Health Sciences, Spokane, WA 99202 USA; 6 Colgate Palmolive Company, Piscataway, NJ 08854 USA

**Keywords:** Semi-synthetic-sulfated-polysaccharide, Inflammation, Cytokines, Matrix metalloproteinases (MMPs), Periodontitis

## Abstract

**Background:**

Chronic periodontitis is associated with an increased risk for systemic conditions such as cardiovascular disease, diabetes, and osteoporosis. During chronic periodontitis, endotoxin (lipopolysaccharide, LPS) produced by *P. gingivalis* provokes monocyte accumulation and differentiation into macrophages and increased secretion of pro-inflammatory cytokines and matrix metalloproteases (MMPs). While normal levels of MMPs are important in cellular function, increased levels of cytokines and MMPs can cause connective tissue destruction.

**Results:**

In the current study, we investigated the therapeutic capability of a novel semi-synthetic sulfated polysaccharide (SAGE) on the production of cytokines and MMPs by cultured human mononuclear cells and macrophages stimulated with endotoxin LPS produced by *P. gingivalis*, a periodontally-relevant cell culture model. Our research demonstrated SAGE inhibited the LPS induced synthesis of inflammatory mediators including TNF-α, IL-1β, PGE_2_, and MMP-9 in this periodontal-relevant cell culture model. In addition, TLR-2 and TLR-4 levels were also reduced with the SAGE treatment.

**Conclusions:**

The therapeutic potential of this novel semi-synthetic sulfated polysaccharide compound may help to prevent tissue damage and bone loss in patients with periodontal disease or other inflammatory diseases.

## Background

Periodontal disease is the most prevalent chronic inflammatory diseases characterized by recurrent bacterial infection followed by immune responses derived from host which often progresses into connective tissue breakdown and alveolar bone loss [1]. During the pathogenesis of periodontitis, anaerobic gram-negative periodontal-associated pathogens (e.g., *P. gingivalis, T.forsythia*) and the lipopolysaccharide (LPS, endotoxin) in their cell walls stimulate the innate and adaptive immune responses in periodontal tissues [2]. LPS and other virulence factors stimulate the host immune-inflammatory response by binding to Toll-like receptors 2 (TLR-2) and 4 (TLR-4). Both TLR-2 and TLR-4 are membrane proteins, express excessively in peripheral blood leukocytes such as neutrophils and monocytes. TLR-4 is most well-known for recognizing LPS during periodontal inflammation, and play a fundamental role in pathogen recognition and activation of innate immunity via stimulation of NF-κB [3]. This results in local accumulation of inflammatory cells such as neutrophils and monocytes/macrophages and generate elevated levels of cytokines and other proinflammatory mediators such as the prostaglandins. These mediators exert autocrine or paracrine activities by upregulating the expression of matrix metalloproteinase (MMP) expression and in turn its activity contributes to the destruction and loss of periodontal connective tissue [4].

**Fig. 1:**
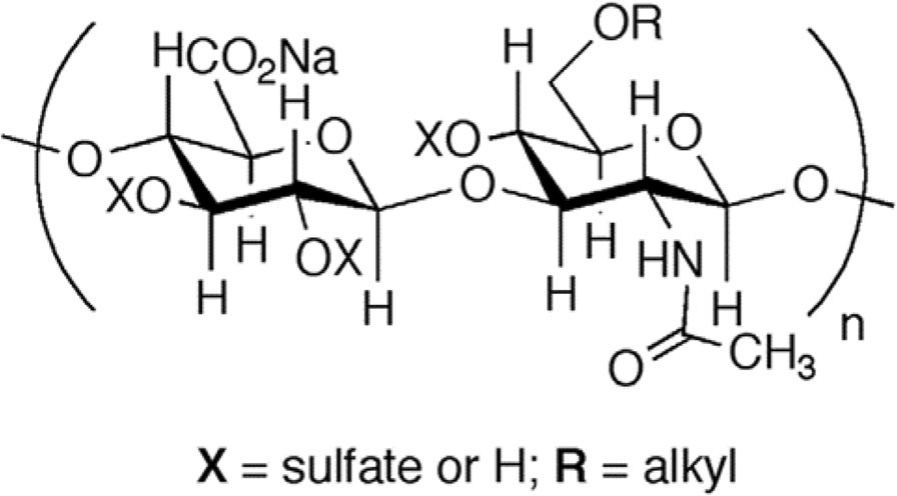
Structure of semi-synthetic glycosaminoglycan ethers (SAGEs)

A family of sulfated and metabolically stabilized anionic polysaccharide derivatives known as semi-synthetic glycosaminoglycan ethers (SAGEs) have been developed and their therapeutic potential to treat chronic inflammatory diseases such as periodontitis have been evaluated [5]. SAGE is synthesized from sulfating and alkylation of non-animal derived hyaluronic acid (HA), an immunoneutral skin polysaccharide consisting of long polymers of the disaccharide N-acetylglucosamine (GlcNAc) and glucuronic acid (GlcA) linked GlcNacb1-3GlcAb1–4 in repeating units along the chain. A representative SAGE structure is illustrated in (Fig. 1) which was produced from 53 kDa HA and had a final molecular weight of 5.5 kDa.

It has been shown that SAGE exhibits substantial anti-inflammatory activities at nanomolar concentrations, including inhibition of cationic PMN proteases, inhibition of the leukocyte adhesion receptor P-selectin, and inhibition of the interaction of the receptor for advanced glycation end-products (RAGE) with its disparate ligands [6]. Additionally, SAGE effectively reduce inflammation in an animal model of rosacea by binding to cationic cathelicidin peptide LL-37 [7]. Since chronic inflammatory condition such as periodontitis is characterized by a local accumulation of leukocytes, predominantly (70%) mononuclear cells, in the present study, we investigated the effect of a novel semi-synthetic sulfated polysaccharide (SAGE) on the production of cytokines and MMPs by cultured human mononuclear cells/macrophages stimulated with endotoxin (lipopolysaccharide, LPS) produced by *P. gingivalis*, a periodontally-relevant cell culture model.

## Results

### Cell culture studies - human peripheral blood mononuclear cells (PBMC)

**Fig. 2:**
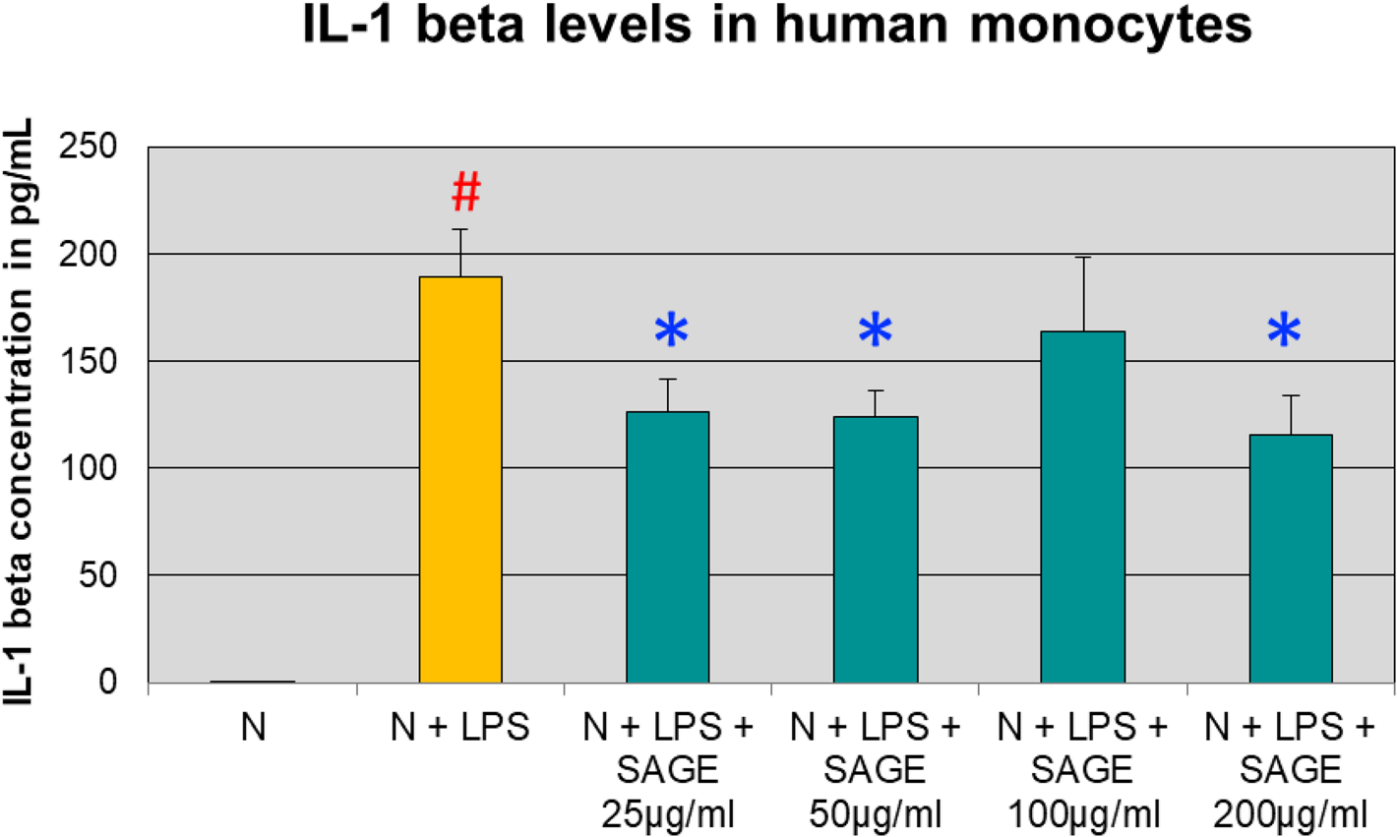
Inhibition of IL-1β levels by SAGE in human monocytes. Monocytes (1 × 10^6^ cells/well) were cultured in serum-free media (37 °C, 5% CO_2_/95%O_2_ 18 h) with LPS (50 ng/mL), SAGE, or vehicle alone. Conditioned medium were analyzed for IL-1β by ELISA. Each value represents the mean of 3 cultures ± the standard error of the mean (S.E.M.). #: *p* < 0.05 vs normal and *: *P* < 0.05 vs LPS

As shown in Fig. 2, monocytes from human peripheral blood produced minimal levels of IL-1β (< 25 pg/ml). When LPS (50 ng/mL) was added to the culture, monocytes secreted 189 pg/mL of IL-1β with 99.9% significant increase (*P* < 0.05). SAGE at different concentrations (25 μg/ml to 200 μg/ml) significantly reduced IL-1β levels (*P* < 0.05) by 35% approximately when stimulated with LPS.

**Fig. 3:**
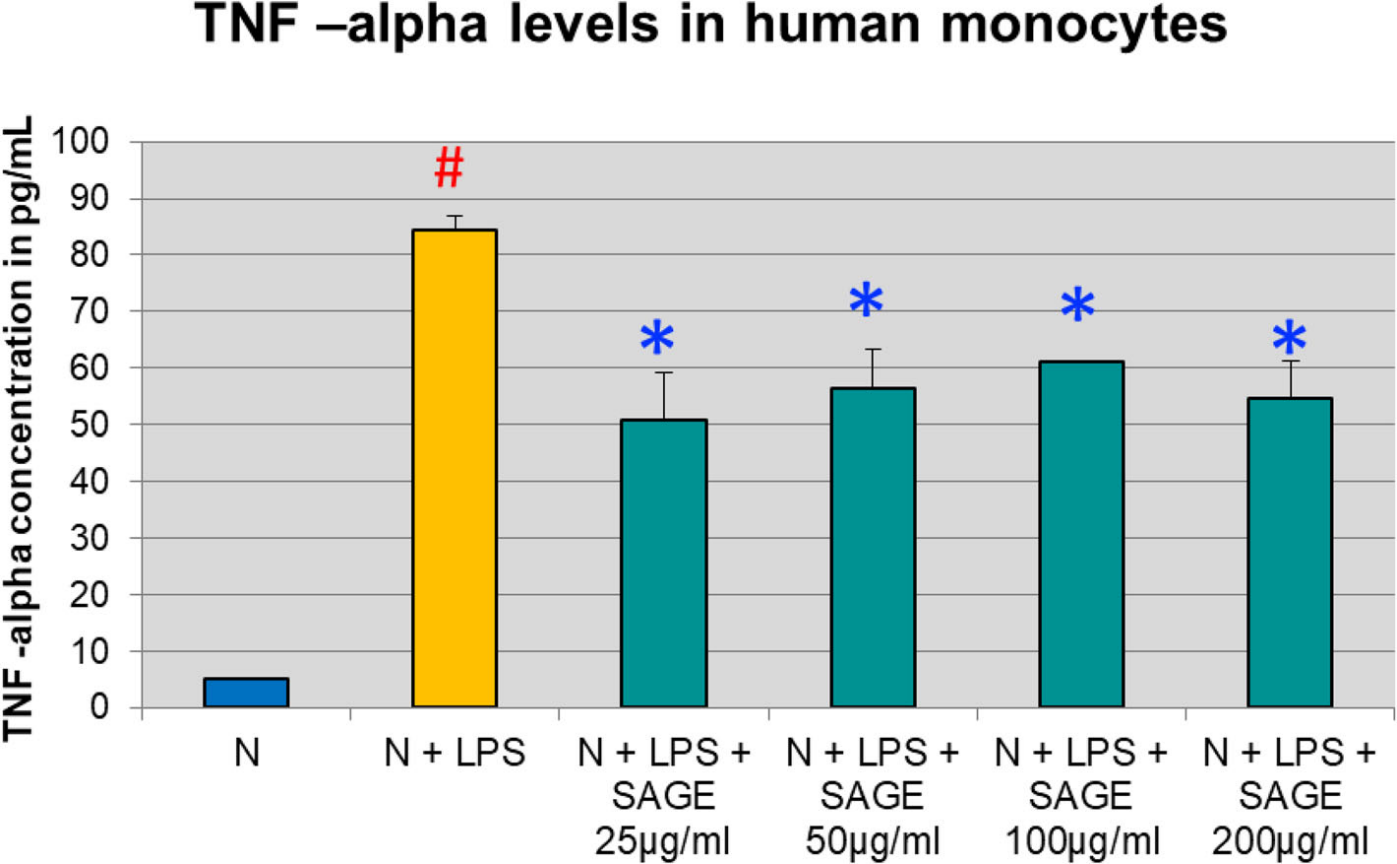
Inhibition of TNF-α levels by SAGE in human monocytes. Monocytes (1 × 10^6^ cells/well) were cultured in serum-free media (37 °C, 5% CO_2_/95%O_2_ 18 h) with LPS (50 ng/mL), SAGE, or vehicle alone. Conditioned medium were analyzed for TNF-α by ELISA. Each value represents the mean of 3 cultures ± the standard error of the mean (S.E.M.). #: *p* < 0.05 vs normal and *: *P* < 0.05 vs LPS

Similarly, monocytes secreted 84 pg/mL of TNF -α with 93% significant (*p* < 0.05) increase when LPS was added to the culture, SAGE at different concentrations significantly reduced TNF -α levels (*P* < 0.05) by 35% approximately when stimulated with LPS (Fig. 3).

PGE_2_ and IL-6 levels were evaluated as well. Both inflammatory mediators were significantly increased when monocytes were stimulated with LPS. PGE_2_ level was increased from 0.47 ng/mL to 0.9 ng/mL, and IL-6 level was increased from 4 pg/mL to 1812 pg/mL. However, SAGE at different concentrations did not significantly reduce these elevated levels.

### Cell culture studies - human macrophage

**Fig. 4:**
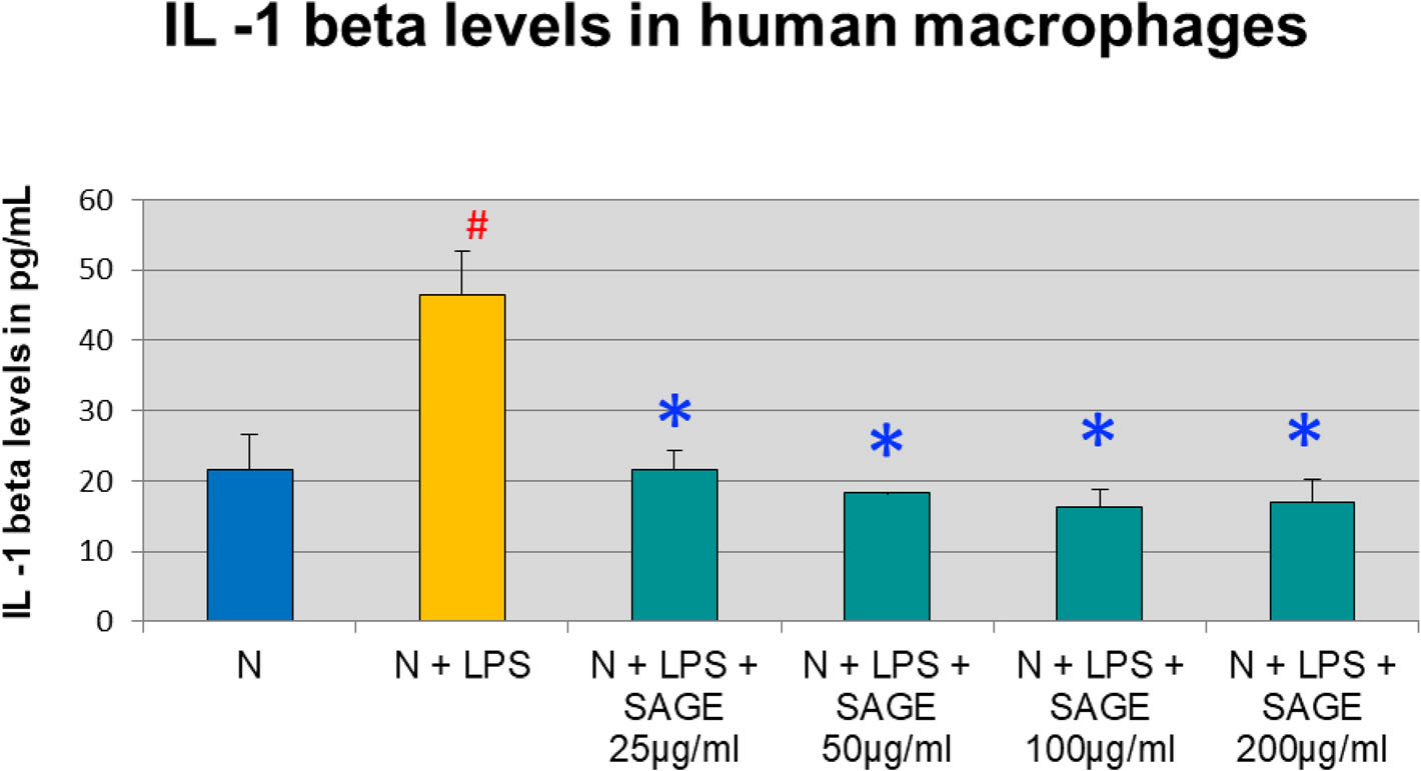
Inhibition of IL-1β levels by SAGE in human macrophages. Macrophages (1 × 10^6^ cells/well) were cultured in serum-free media (37 °C, 5% CO_2_/95%O_2_ 18 h) with LPS (50 ng/mL), SAGE, or vehicle alone. Conditioned medium were analyzed for IL-1β by ELISA. Each value represents the mean of 3 cultures ± the standard error of the mean (S.E.M.). #: *p* < 0.05 vs normal and *: *P* < 0.05 vs LPS

In separate experiments, the monocytes were allowed to mature for 7 days into macrophages and cytokines levels in the CM were assessed by ELISA. Macrophages secreted 46 pg/mL of IL-1β when LPS was added to the culture compared to control cells, a 50% significant increase. SAGE at different concentrations significantly reduced IL-1β levels (*P* < 0.05) by 60% approximately when stimulated with LPS (Fig. 4).

**Fig. 5:**
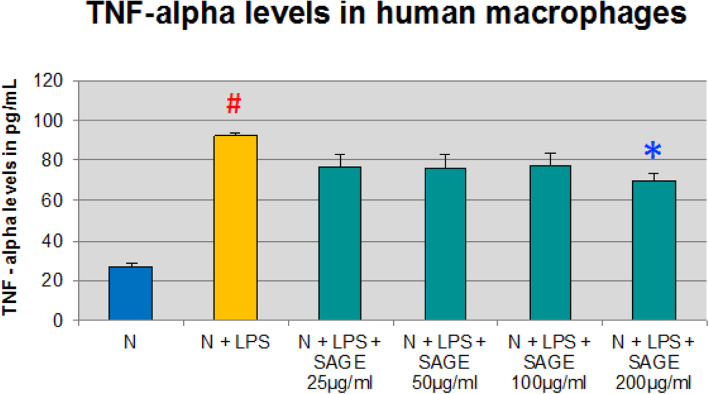
Inhibition of TNF-α levels by SAGE in human macrophages. Macrophages (1 × 10^6^ cells/well) were cultured in serum-free media (37 °C, 5% CO_2_/95%O_2_ 18 h) with LPS (50 ng/mL), SAGE, or vehicle alone. Conditioned medium were analyzed for TNF-α by ELISA. Each value represents the mean of 3 cultures ± the standard error of the mean (S.E.M.). #: *p* < 0.05 vs normal and *: *P* < 0.05 vs LPS

Similarly, macrophages alone secreted 27 pg/mL of TNF-α, When LPS was added to the culture, TNF-α level was increased significantly by 70% (*p* < 0.05). SAGE at final concentration of 200 μg/ml significantly reduced TNF -α levels (*P* < 0.05), and SAGE also reduced TNF-α levels by 20% approximately with other concentrations when stimulated with LPS (Fig. 5), but was not statistically significant.

**Fig. 6:**
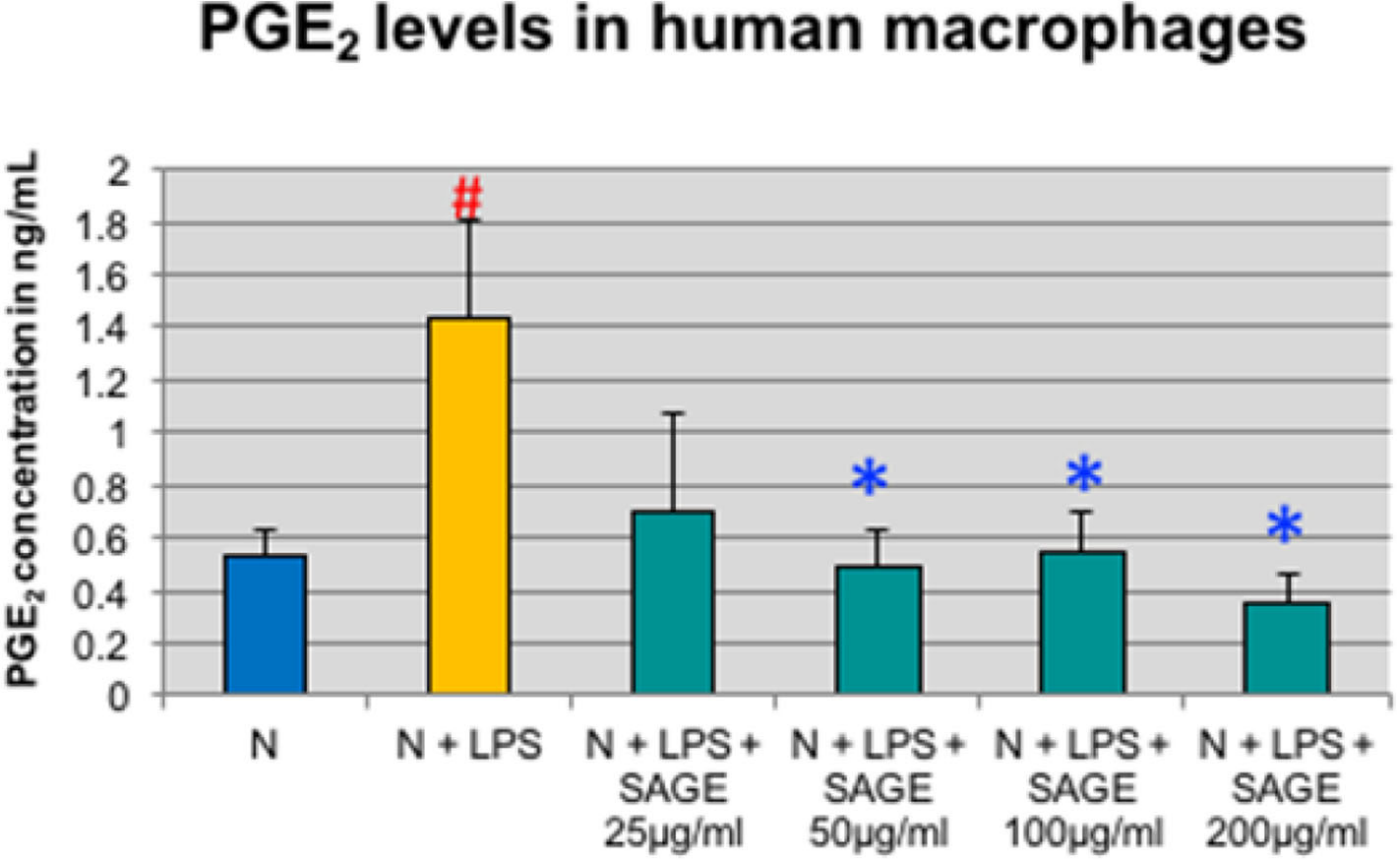
Inhibition of PGE_2_ levels by SAGE in human macrophages. Macrophages (1 × 10^6^ cells/well) were cultured in serum-free media (37 °C, 5% CO_2_/95%O_2_ 18 h) with LPS (50 ng/mL), SAGE, or vehicle alone. Conditioned medium were analyzed for PGE_2_ by ELISA. Each value represents the mean of 3 cultures ± the standard error of the mean (S.E.M.). #: *p* < 0.05 vs normal and *: *P* < 0.05 vs LPS

In contrast to monocytes, PGE_2_ level was significantly increased by 170% when macrophages were stimulated with LPS (*p* < 0.05) and there was significant reduction in PGE_2_ levels in the presence 200 μg/ml SAGE and there was approximately 60% reduction in presence of 25 to 100 μg/ml of SAGE (Fig. 6).

The macrophages stimulated with endotoxin did not lead to significant elevations in IL-6 above un-stimulated cells and slight reduction of IL-6 levels was seen at 100–200 μg/ml SAGE, but was not statistically significant.

**Fig. 7:**
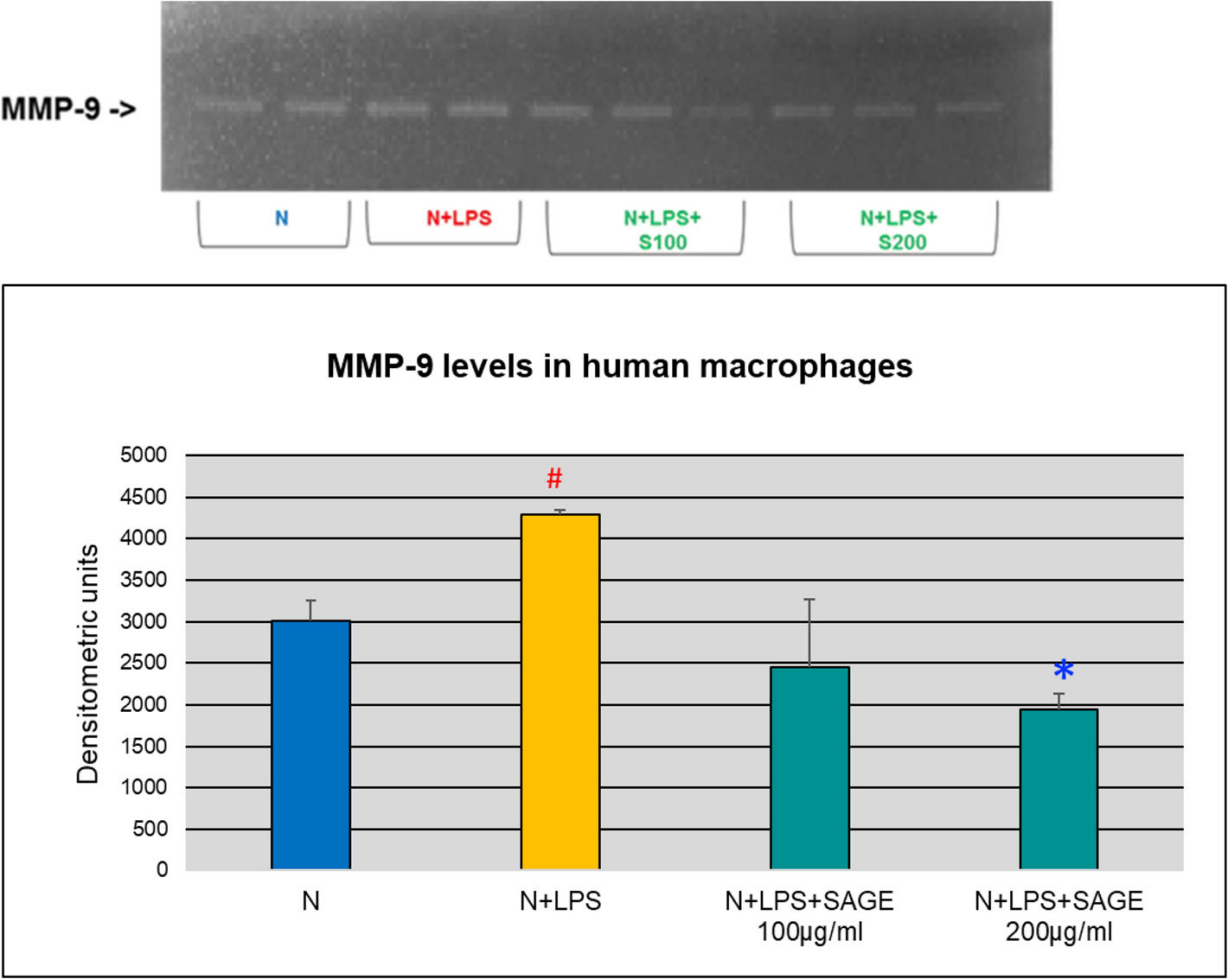
Inhibition of MMP-9 levels by SAGE in human macrophages (gelatin zymography). Macrophages (1 × 10^6^ cells/well) were cultured in serum-free media (37 °C, 5% CO_2_/95%O_2_ 18 h) with LPS (50 ng/mL), SAGE, or vehicle alone. Conditioned medium were analyzed by gelatin zymography. Levels of MMP-9 and MMP-2 were quantified by measuring band intensity using Image J. Each value represents the mean of 3 cultures ± the standard error of the mean (S.E.M.). #: *p* < 0.05 vs normal and *: *P* < 0.05 vs LPS

**Fig. 8:**
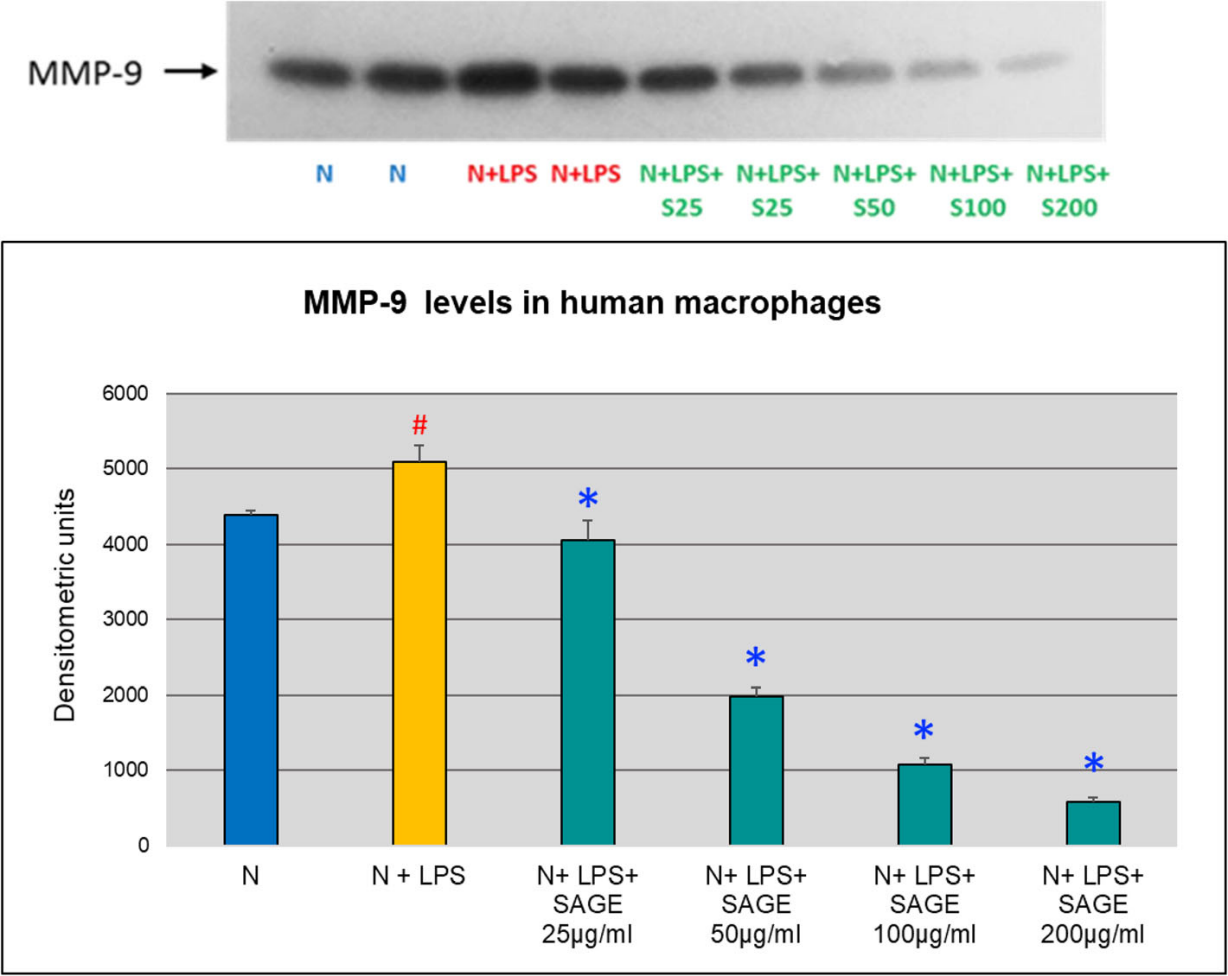
Inhibition of MMP-9 levels by SAGE in human macrophages (Western blot). Macrophages (1 × 10^6^ cells/well) were cultured in serum-free media (37 °C, 5% CO_2_/95%O_2_ 18 h) with LPS (50 ng/mL), SAGE, or vehicle alone. Conditioned medium were analyzed by Western blot. Levels of MMP-9 and MMP-2 were quantified by measuring band intensity using Image J. Each value represents the mean of 3 cultures ± the standard error of the mean (S.E.M.). #: *p* < 0.05 vs normal and *: *P* < 0.05 vs LPS

MMP-9 and MMP-2 levels in macrophage CM were also evaluated by gelatin zymography and western blot. When MMP-9 levels were quantified, there was significant increase (*p* < 0.05) in MMP-9 levels in the conditioned media stimulated with LPS. SAGE at different concentrations significantly reduced (*p* < 0.05) MMP-9 levels by 70% approximately in a dose-dependent manner (Figs. 7 and 8). No significant MMP-2 levels were detected by gelatin zymography.

**Fig. 9:**
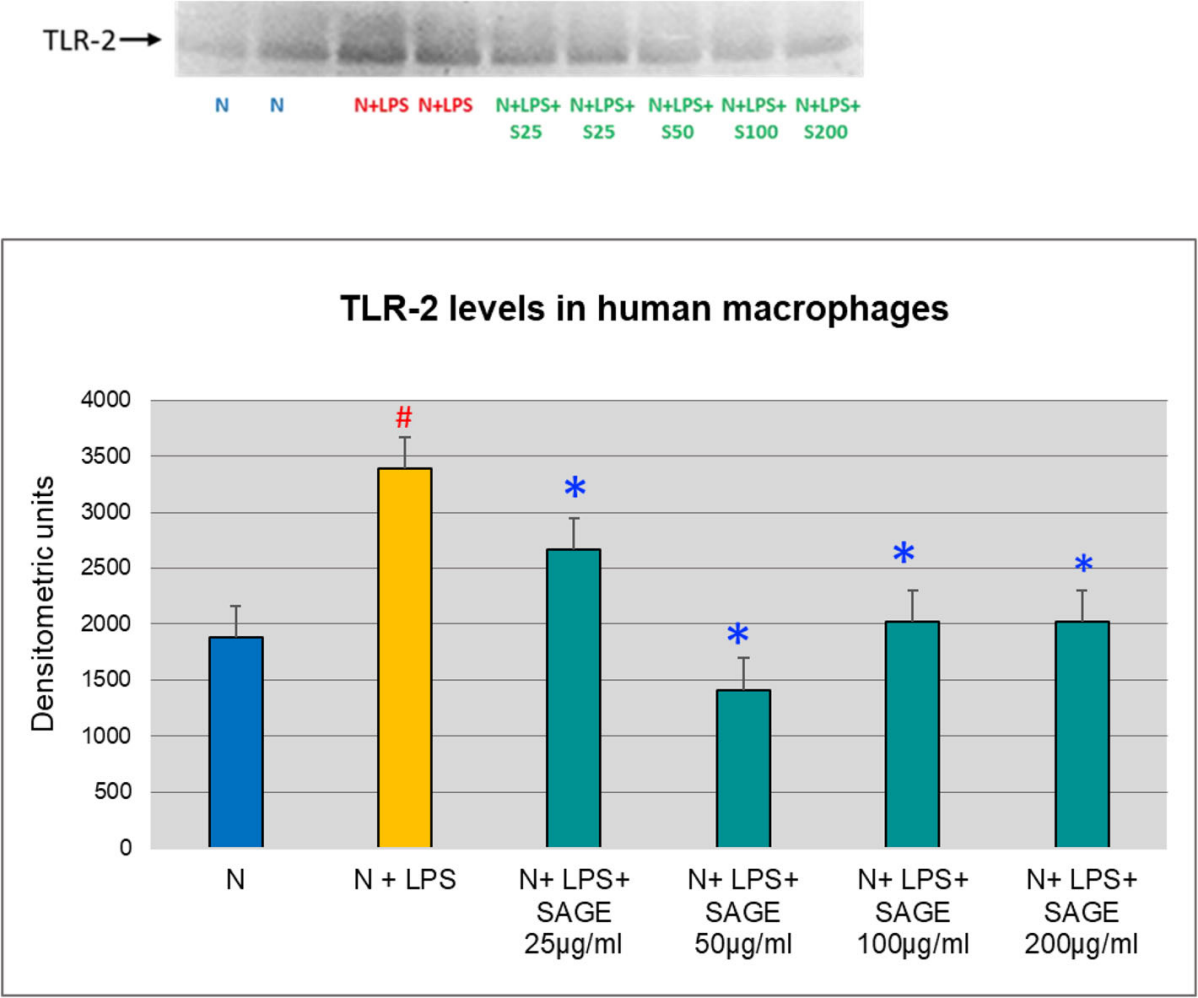
Inhibition of TLR-2 levels by SAGE in human macrophages (Western blot). Macrophages (1 × 10^6^ cells/well) were cultured in serum-free media (37 °C, 5% CO_2_/95%O_2_ 18 h) with LPS (50 ng/mL), SAGE, or vehicle alone. Conditioned medium were analyzed by Western blot. Levels of TLR-2 were quantified by measuring band intensity using Image J. Each value represents the mean of 3 cultures ± the standard error of the mean (S.E.M.). #: *p* < 0.05 vs normal and *: *P* < 0.05 vs LPS.

**Fig. 10:**
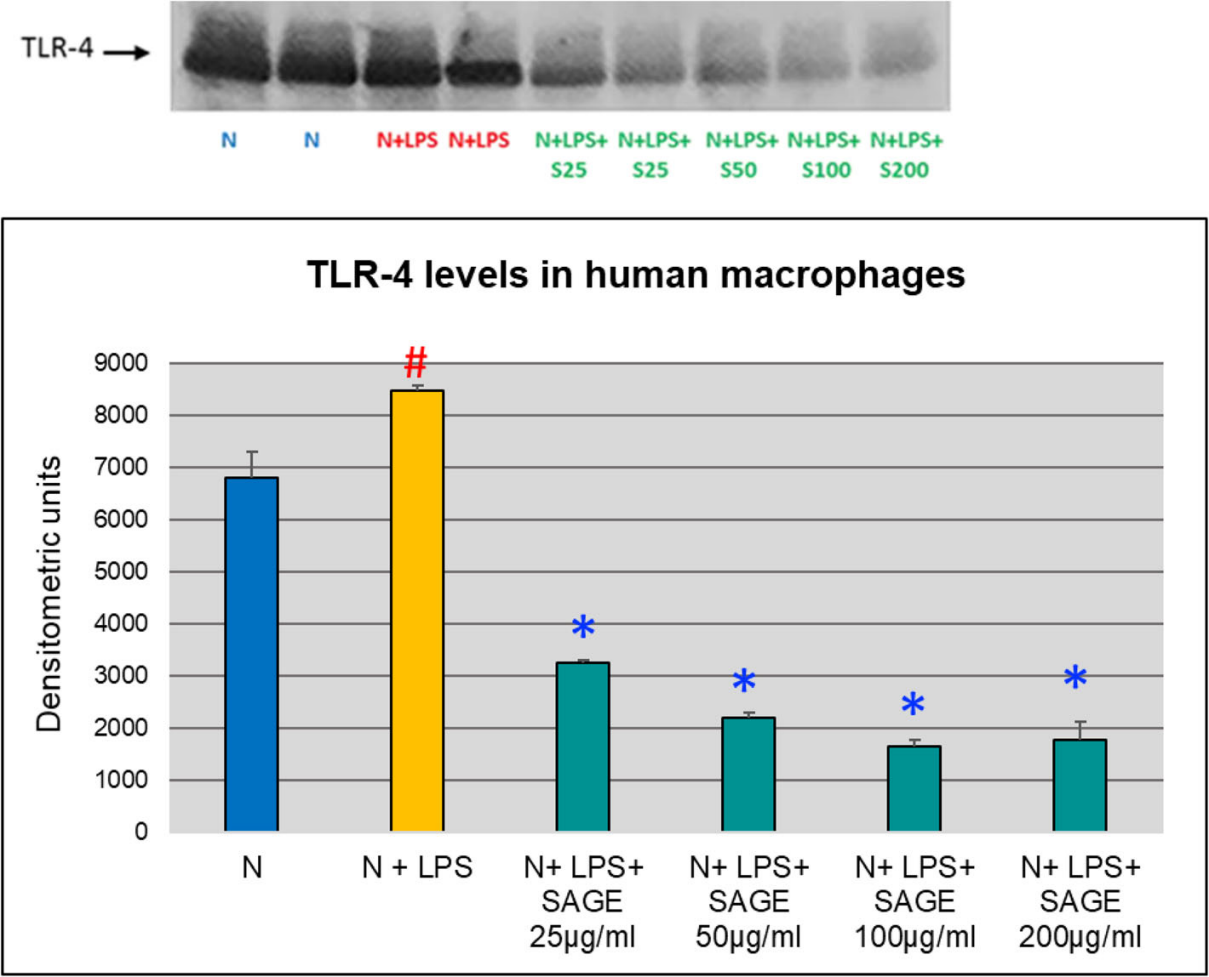
Inhibition of TLR-4 levels by SAGE in human macrophages (Western blot). Macrophages (1 × 10^6^ cells/well) were cultured in serum-free media (37 °C, 5% CO_2_/95%O_2_ 18 h) with LPS (50 ng/mL), SAGE, or vehicle alone. Conditioned medium were analyzed by Western blot. Levels of TLR-4 were quantified by measuring band intensity using Image J. Each value represents the mean of 3 cultures ± the standard error of the mean (S.E.M.). #: *p* < 0.05 vs normal and *: *P* < 0.05 vs LPS

Both TLR-2 and TLR-4 play essential role in periodontal inflammation. Therefore, to begin to explore the underlying mechanism of SAGE on its inhibitory effects on the inflammatory mediators, the effects of SAGE on TLR-2 and TLR-4 levels in monocyte-derived macrophage culture supernatants were analyzed by Western blotting. Both TLR-2 and TLR-4 were detected. The band density showed 80% significant increase (*p* < 0.05) of TLR-2 and TLR − 4 levels when macrophages were stimulated with LPS and there is 50% significant decrease (*p* < 0.05) in TLR-2 levels with different concentrations of SAGE when stimulated with LPS. Similar effect was seen with TLR-4 levels where SAGE at different concentrations significantly reduced (*p* < 0.05) TLR-4 levels and there was approximately 80% reduction (Figs. 9 and 10).

**Fig. 11:**
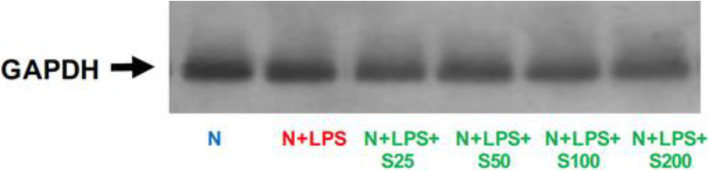
GAPDH levels in human macrophages. Macrophages (1 × 10^6^ cells/well) were cultured in serum-free media (37 °C, 5% CO_2_/95%O_2_ 18 h) with LPS (50 ng/mL), SAGE, or vehicle alone. Levels of GAPDH in conditioned medium were by Western blot

GAPDH levels were used as internal controls and there was no difference in band density in human macrophage supernatants (Fig. 11).

## Discussion

**Fig. 12:**
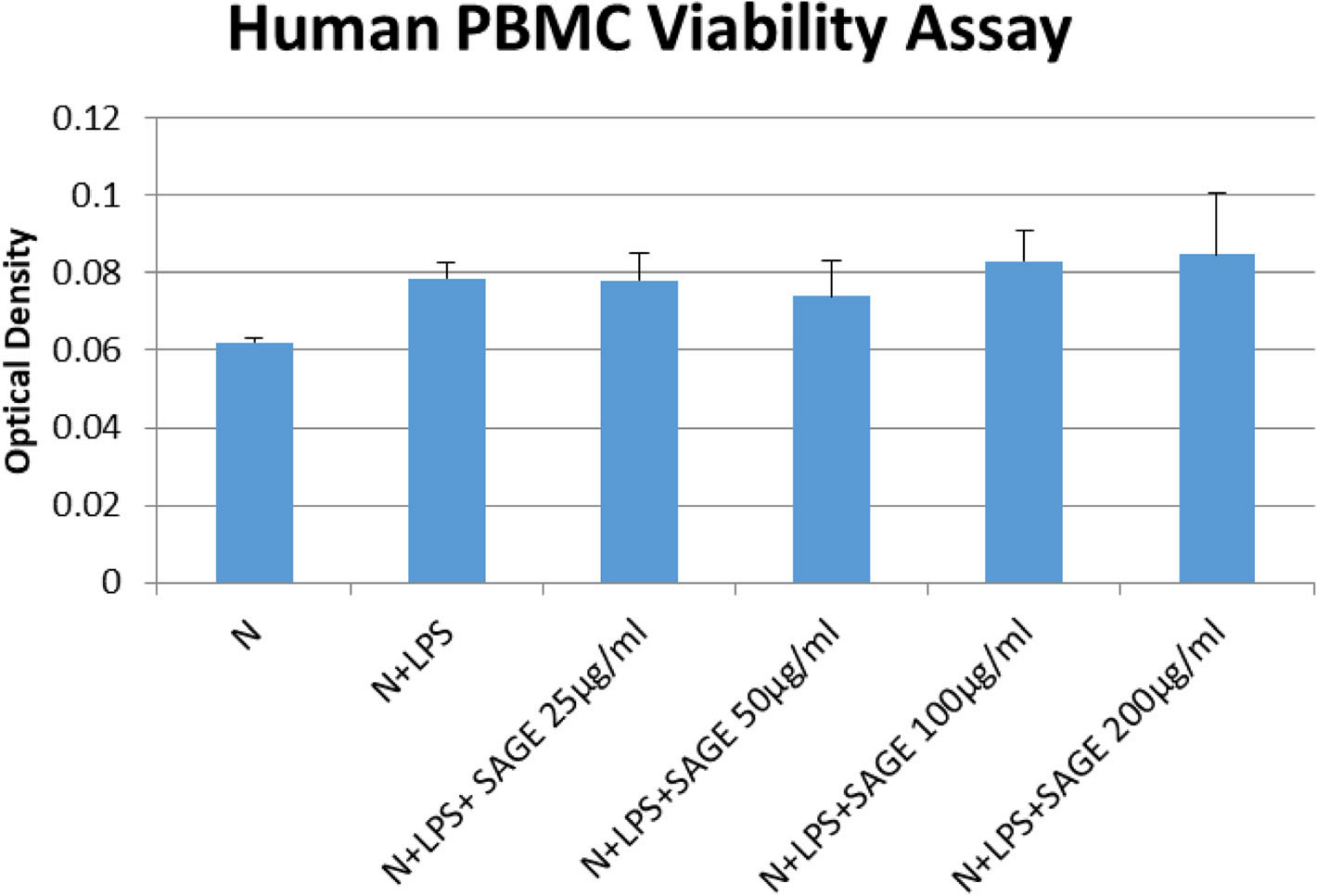
Human PBMC MTS viability assay. Human PBMC (1 × 10^6^ cells/well) were cultured in serum-free media (37 °C, 5% CO_2_/95%O_2_ 18 h) with LPS (50 ng/mL), SAGE, or vehicle alone. MTS tetrazolium compound was added and incubated for 2 h. The formazan dye was quantified by measuring the absorbance at 490 nm. Each value represents the mean of 3 cultures ± the standard error of the mean (S.E.M.)

Periodontitis is a chronic inflammatory disease where the host modulatory response plays a critical role in inflammation. Mononuclear phagocytes are attracted to the site of inflammation by chemotactic signals which in turn releases proteinases and cytokines resulting in tissue damage. Previous studies showed that SAGE, highly sulfated GAG is anti-inflammatory at nano-molar concentration including blockade of P- and L-selectin, inhibition of PMN derived human neutrophil elastase (HLE) and attenuates LL-37 induced bladder inflammation. In addition, Glycosaminoglycans (GAGs), such as hyaluronic acid, heparin, and more recently synthetic GAGs, have been shown to interfere with bacterial growth. SAGEs are bacteriostatic, potently inhibit CR3, block CR3-mediated *Pg* uptake in human macrophages [5, 8]. Herein we show that this novel sulfated glysoaminoglycan ether has broad anti-inflammatory activity. When SAGE was added to the monocyte derived macrophages in cell cultures at 25–200 μg/ml, it attenuated upstream inflammatory signaling receptors, pro-inflammatory cytokines as well as matrix metalloproteinases. Our data in most assays indicated that the maximum inhibition was achieved by the lowest concentration 25 μg/ml applied, the inhibitory effects at higher concentrations of SAGE were plateaued. This observation is taken into consideration when evaluating in vivo doses. The cell cytotoxicity was also evaluated to exclude that the anti-inflammatory effect of SAGE was not the result of cell toxicity (Fig. 12). A MTS assay with a tetrazolium compound was used to assess cell proliferation, cell viability and cytotoxicity. The results showed that SAGE at concentrations from 25 to 200 μg/ml was not toxic to human peripheral blood mononuclear cells (PBMC).

The initial driving force for periodontitis is infection of the gingival tissues with oral bacteria, particularly those of the anaerobic, gram-negative “red complex”, primary of which is *P. gingivalis* [9]. This bacterial biofilm is the primary inflammatory stimulus for chronic inflammation, however, the ultimate outcome of the disease is determined by the host response. Recognition of pathogenic bacteria by the host requires mechanisms to mount an appropriate response that can prevent the dissemination of infection. The host immune system detects invading pathogens primarily through pattern-recognition receptors. These receptors recognize pathogen associated molecular patterns (PAMPs) that are typically shared by large groups of microorganisms, i.e. LPS [10]. The Toll-like receptors are members of interleukin-1 super-family of transmembrane receptors that identify pathogen associated molecular patterns [11]. For the recognition and transduction of the lipopolysaccharide signal the transmembrane Toll-like receptor-4 and 2 present in the mononuclear phagocytes plays a pivotal role. Upon binding of lipopolysaccharide, the Toll-like receptor-4 activates the inflammatory cascade system ultimately leading to the synthesis and release of pro-inflammatory cytokines and proteinases which are potential stimulators for tissue destructive effects in periodontitis [12]. Our Immunoblotting results showed the expression levels of TLR-2 and TLR- 4 in macrophages were diminished with 25–200 μg/ml of SAGE, which can contribute to the downstream reduction in the levels of pro-inflammatory mediators.

Pro-inflammatory cytokines such as IL-1β, TNF-α and IL-6 are produced by monocytes, and macrophages and play a critical role in the pathogenesis of periodontitis [13–15]. Once these cytokines are released, they stimulate the production of critical enzymes such as MMPs, which are largely responsible for the direct breakdown of connective tissues in chronic inflammatory diseases [16, 17]. IL-1β and TNF-α and IL-6 also activate other mediators of inflammation such as cycloxygenase-2 and 5- lipoxygenase, leading to the production of lipid mediators of inflammation, such as prostaglandins (PGE_2_) and leukotrienes [18]. Inhibition of pro-inflammatory cytokines decreases the loss of connective tissue attachment thereby attenuating periodontitis. SAGE has been found to exhibit anti- inflammatory activities. In this study SAGE effectively reduced the levels of IL-1β and TNF-α in monocyte significantly whereas there was not much effect on systemic cytokines IL-6 and PGE_2_. While in cell culture assays with human macrophages, there was dose-dependent decrease in levels of IL-1β, TNF- α and PGE_2_ which in turn reduces the MMP-9 levels thereby inhibiting the host-mediated inflammatory cascade system. SAGE disrupts the events associated with periodontitis in multiple manners,  spanning from the early pro- inflammatory TLR signaling to molecular pathways involving pro-inflammatory cytokines and MMPs, and consequently prevents tissue breakdown.

## Conclusions

In conclusion, the findings presented in this paper demonstrated the cell-mediated anti-inflammatory activity of SAGE in the treatment of chronic inflammatory diseases such as periodontal disease, is mediated at least in part through TLR-2 and TLR-4 signaling pathway. The suppression of inflammatory signaling and MMPs can be beneficial to reduce pathologically excessive degradation of the extracellular matrix including connective tissues and alveolar bones. Currently, the anti-inflammatory effect of SAGE in the management of periodontal disease is being investigated in vivo in a diabetic rat model. The results of the in vivo research will be reported in a future article.

## Materials and methods

### Chemical reagents

All chemical reagents, and LPS from *P. gingivalis* were purchased from Sigma-Aldrich Co. (St. Louis, MO). Cell culture reagents were purchased from Gibco/Invitrogen Corp. (Carlsbad, CA). SAGE (GM − 1111) was obtained from GlycoMira, LLC (Salt Lake City, UT).

### Cell culture assay

Human peripheral blood mononuclear cells (PBMC) were isolated and purified from Leukocyte Concentrate, which was purchased from Long Island Blood Bank (Melville, NY). The project (2007–6548) was approved for exemption by Stony Brook University Committees on Research Involving Human Subjects (CORIHS). Human PBMC were isolated by density gradient centrifugation as described by us previously [19]. PMBC were cultured in serum-free macrophage media (Invitrogen Corp, Carlsbad, CA) for 18 h. LPS from *P. gingivalis* (50 ng/mL) or vehicle were added before the incubation. SAGE was added at final concentrations of 25 to 200 μg/ml. Conditioned media (CM) were analyzed for the cytokines and pro-inflammatory mediators, Tumor necrosis factor – alpha (TNF-α), Interleukin − 1 beta (IL-1β), Interleukin − 6 (IL-6), and Prostaglandin E_2_ (PGE_2_), by enzyme-linked immunosorbent assay (ELISA) as described by us previously [20].

In separate assays, PBMC were cultured for 7 days to allow for maturation to macrophage. Macrophages were then cultured for 18 h with or without LPS (50 ng/mL). SAGE at different concentrations were also added to the culture at the beginning of the experiment. The CM were analyzed for 1. TNF-α, IL-1β, IL-6 and PGE_2_ by ELISA; 2. MMPs by gelatin zymography and Western blot; and 3. TLR-2 and TLR-4 expression by Western blot. Gelatin zymography and Western blot assays were described below.

### MTS cell viability assay

Human PBMC (1 × 10^6^ cells/well) were cultured in serum-free media (37 °C, 5% CO_2_/95%O_2_ 18 h) with LPS (50 ng/mL), SAGE, or vehicle alone. The CellTiter 96® AQueous One Solution Reagent contains a tetrazolium compound was added to the culture and incubated for 2 h. The formazan product was quantified by measuring the absorbance at 490 nm. The MTS assay kit was purchased from Promega Corp. (Madison, WI).

### ELISA assay

Cell culture CM collected from the above assays were analyzed for pro-inflammatory cytokines: TNF-α, IL-1β, IL-6 and PGE_2_. ELISA kits for TNF-α, IL-1β, IL-6 and PGE_2_, were purchased from R&D Systems, Inc. (Minneapolis, MN). ELISA assays were performed and followed manufacturer’s protocol.

### Gelatin Zymography

The gelatin zymography system were purchased from Invitrogen Corp. (Carlsbad, CA). MMP-2 and MMP-9 standards were purchased from R&D Systems, Inc. (Minneapolis, MN). CM collected from the above cell culture assays were analyzed by gelatin zymography as described by us previously [20]. SDS-PAGE gels with 1 mg/ml gelatin were used. After electrophoresis, the gels were washed with 2.5% Triton X-100 and incubated at 37 °C overnight in calcium assay buffer (40 mM Tris/HCl, 200 mM NaCl, 10 mM CaCl2, pH 7.5), then stained with Coomassie Brilliant Blue R-250. As described by us earlier [19], clear zones of lysis against a blue background indicate gelatinolytic activity and levels of MMP-9 and MMP-2 were quantified by measuring band intensity using Image J analysis software [21].

### Western blot analysis

CM collected from human macrophage cell culture were analyzed for TLR-2, TLR-4, and MMP-9 by Western blot analysis. All Western blot procedures were performed as described by us previously [22]. Prestained molecular weight markers were purchased from Bio-Rad Laboratories, Inc. (Hercules, CA). CM samples were electrophoresed on SDS-PAGE (8% separating and 4% stacking gels). Proteins were transferred to nitrocellulose membranes, and incubated with primary antibodies (TLR-2: Abcam, Cambridge, MA; TLR-4: Life Technologies, Carlsbad, CA; MMP-9: Cell Signaling Technology, Danvers, MA). Excess primary antibodies were removed before incubation with the secondary antibodies conjugated to horseradish peroxidase (Goat anti-rabbit antibodies, Cell Signaling Co., Danvers, MA). Detection of the protein bands was carried out by scanning the membranes with Invitrogen™ iBright™ FL1000 Western Blot Imaging Systems (Thermo Fisher Scientific, Inc., USA) and quantified by measuring band intensity using Image J analysis software. Glyceraldehyde-3-phosphate dehydrogenase (GAPDH) was used as an internal control.

### Statistical analysis

Cytokine, MMP and receptor levels between groups were analyzed by analysis of variance (ANOVA), with *P* ≤ 0.05 taken as statistically significant. Normal versus LPS is represented by #; LPS versus SAGE treatment is represented by *.

## Data Availability

The data generated and/or analysed during the current study are available from the corresponding author on reasonable request with the permission from GlycoMira Therapeutics, Inc.
